# Propensity score matching analysis of the relationship between allogeneic blood transfusion and postoperative pulmonary complications in scoliosis correction surgery: a retrospective study

**DOI:** 10.3389/fmed.2025.1588218

**Published:** 2025-10-14

**Authors:** Qi Gao, Tingting Wang, Junyu Zhou, Ruiyu Wang, Yang Yu, Zexin Chen, Yuexiu Chen, Jingcheng Zou, Linqian Zhao, Yuanyuan Yao, Bin Zheng, Min Yan

**Affiliations:** ^1^Department of Anesthesiology, The Second Affiliated Hospital, Zhejiang University School of Medicine, Hangzhou, China; ^2^Department of Anesthesiology, Weifang People’s Hospital, Weifang, Shandong, China; ^3^Statistician Center of Clinical Epidemiology & Biostatistics, Department of Scientific Research, The Second Affiliated Hospital, School of Medicine, Zhejiang University, Zhejiang, China; ^4^Department of Surgery, University of Alberta, Edmonton, AB, Canada; ^5^Zhejiang Key Laboratory of Pain Perception and Neuromodulation, Hangzhou, China

**Keywords:** spine, transfusion, postoperative pulmonary complications, scoliosis, patient blood management, propensity score matching analysis

## Abstract

**Background:**

Surgery is still the treatment of choice for patients with moderate to severe scoliosis, and vertebral column resection can significantly correct scoliosis. However, scoliosis correction surgery is associated with a high incidence of perioperative complications. We hypothesize that receiving allogeneic blood transfusions during surgery increases the risk of these complications in patients undergoing scoliosis correction surgery.

**Methods:**

This retrospective study included 512 patients who underwent scoliosis correction surgery at the Second Hospital of Zhejiang University School of Medicine between August 2016 and April 2023. Patients who experienced or did not experience transfusion were balanced in terms of baseline clinicodemographic characteristics using propensity score matching. Multivariable logistic regression of the balanced data was performed to assess the potential influence of intraoperative allogeneic transfusion on incidence of PPCs.

**Results:**

Propensity score matching led to a dataset of 322 patients, of whom 161 experienced allogeneic transfusion and 161 did not. Multifactorial logistic regression identified the following factors associated with PPCs: intraoperative allogeneic red blood cell transfusion rate (Risk Ratio (RR) 1.53 95% confidence interval (CI) 1.12–2.11, *p* = 0.007). The risk of PPCs increased with increasing volume of allogeneic blood transfusions, with those receiving 400 mL and more being at greater risk compared to those receiving no more than 400 mL (RR 1.40, 95% CI 1.04–1.89, *p* = 0.030). Subgroup analyses showed increased PPC risk in females, longer surgeries (>3 h), and patients without TXA use.

**Conclusion:**

Intraoperative allogeneic red blood cell transfusion rate and volume during scoliosis correction surgery may be strongly associated with occurrence of PPCs.

## Introduction

Surgery is still the treatment of choice for patients with moderate to severe scoliosis (1, 2), and vertebral column resection can significantly correct scoliosis (3). However, scoliosis correction surgery is associated with a high incidence of perioperative complications (4), including peri- and postoperative pulmonary complications (PPCs), which occur in nearly 4% of patients (5). More than 40% of all deaths after scoliosis correction surgery are attributed to PPCs (6).

PPCs arise from a complex interplay of patient, surgical, and anesthesia-related factors, which are associated with type and duration of surgery (44). Scoliosis correction surgery can result in significant intraoperative bleeding and invisible postoperative blood loss, particularly when the procedure is long or involves vertebral body removal or fusion of multiple segments (7). Approximately 50–80% of patients require perioperative transfusion during scoliosis correction surgery (8), which can increase the risk of postoperative complications and longer hospital stay to an extent proportional to the transfusion volume (9, 10). In patients undergoing elective spine surgery, transfusion of as little as one unit of allogeneic blood can prolong hospital stay and increase risk of postoperative complications (11). Meanwhile, the risks associated with blood transfusion are even greater among patients with severe preoperative anemia (12). It has been shown that allogeneic blood transfusion in elective spine surgery may be associated with increased postoperative complications, length of hospital stay, and rate of 30-day readmission (13). However, whether it influences risk of PPCs is unclear.

Although recent studies (14, 15) have confirmed that perioperative blood transfusions increase the risk of postoperative complications in spinal surgeries, research specifically exploring the association between allogeneic transfusion and postoperative pulmonary complications (PPCs) in scoliosis correction surgery remains relatively limited (16). Our study seeks to contribute further evidence to this important area.

The primary objective of this study is to examine the impact of intraoperative allogeneic blood transfusion on the incidence of postoperative pulmonary complications. We used propensity score matching to control for potential confounding by risk factors of PPCs previously reported for such patients (17–19). We hypothesize that receiving allogeneic blood transfusions during surgery increases the risk of these complications in patients undergoing scoliosis correction surgery.

## Materials and methods

### Study design

This retrospective study was approved by the Ethics Committee of the Second Affiliated Hospital of Zhejiang University School of Medicine (20210951), which waived the requirement for informed consent due to the retrospective nature of the study. This manuscript adheres to the “Strengthening the reporting of observational studies in epidemiology” (STROBE) guidelines^1^.

Inclusion criteria encompassed patients who underwent Posterior vertebral column resection surgery (PVCR) surgery, such as multisegmental thoracic, lumbar, or thoracolumbar spine surgery, at the Second Affiliated Hospital of Zhejiang University School of Medicine from August 2016 to April 2023. Exclusion criteria comprised patients below 18 years old, those who underwent combined anterior-posterior surgery, growth rod adjustment surgery, or spinal tumor surgery.

### Anesthesia, surgery and blood transfusion

We used a standardized anesthetic management strategy reflecting typical practice at our hospital. In brief, anesthesia was achieved using intravenous injection of propofol (2.0 mg/kg), midazolam (0.1 mg/kg), sufentanil (0.4 μg/kg) and muscle relaxant (Rocuronium bromide, 0.6 mg/kg). Anesthesia was maintained by infusion of propofol (4-8 mg/kg/h), remifentanil (0.02–0.5 g/kg/min), inhaled sevoflurane, as well as intermittent, conservative use of Esmeron and sufentanil. All patients, unless they had contraindications, were treated with hemoprotective strategies, such as acute isovolemic hemodilution or autologous blood recycling (Cell Saver 5, Haemonetics Corporation, Braintree, MA, USA). Meanwhile, all patients were ventilated at a tidal volume of 6- 8 mL/kg and positive end-expiratory pressure of 2–5 cm H2O.

Hemoglobin concentrations were measured hourly during surgery, before and after each blood transfusion, and at the physician’s discretion after surgery using the Cobas b 221 system (Roche, Basel, Switzerland). The transfusion strategy for these patients followed the hospital’s standard practice, with a hemoglobin threshold of 9 g/dL, and transfusions were administered when the hemoglobin level dropped below this threshold. Additionally, tranexamic acid (TXA) was used at the surgeon’s discretion and administered intravenously 10–20 min prior to surgery.

### Data collection

Data were collected by study staff trained in data collection, design and completion of case collection forms, statistical design, diagnostic criteria for complications, and management and confidentiality of data. The collected data were independently verified and stored by two investigators not involved in data collection. From the Clinical Research Laboratory, Department of Anaesthesiology, Second Hospital, Zhejiang University, China.

Data were extracted from the electronic medical record system and “Do Care” anesthesia information system at our hospital. Data were collected on baseline clinicodemographic characteristics and perioperative information. Demographic and clinical characteristics of the patients, such as age, gender, body mass index (BMI), smoking, drink, hypertension, diabetes, respiratory diseases, preoperative HB, preoperative albumin (ALB), American Society of Anesthesiologists (ASA) classification, COBB, and TXA, were collected for both groups. Intraoperative data (number of fused segments, hemostatic drug use, acute isotonic hemodilution application, autologous blood transfusion, allogeneic red blood cell transfusion, fluid usage, bleeding, urine output, operation time), and postoperative data (length of hospital stay, hospital costs). Data were also extracted on preoperative ancillary tests, defined as tests conducted during the week prior to surgery. If tests were repeated during that week, only data from the test closest to surgery were used.

### Study outcome

PPCs were defined as one of four events during hospitalization. Pleural effusion was diagnosed when chest X-ray images showed blunted rib-diaphragm angle, blurred ipsilateral diaphragmatic contour in the upright position, displacement of adjacent anatomical structures, or blurred and cloudy thorax on one side in the supine position (20). Atelectasis: Lung opacification with mediastinal shift, hilum or hemidiaphragm shift toward the affected area, with compensatory hyperinflation in adjacent non-atelectatic lung. Pneumothorax: Air in the pleural space with no vascular bed surrounding the visceral pleura (21). Pneumonia CXR with at least one of the following: infiltrate, consolidation, cavitation;plus at least one of the following: fever >38 °C with no other cause, white cell count 12 × 109 litre-1, >70 yr. of age with altered mental status with no other cause;- plus at least two of the following: new purulent/ changed sputum, increased secretions/suctioning, new/worse cough/dyspnoea/tachypnoea, rales/ bronchial breath sounds, worsening gas exchange (22).

### Statistical analysis

We employed propensity score matching in order to reduce the impact of confounders. Based on demographic and clinical characteristics, propensity scores were calculated using logistic regression. We used 1:1 greedy nearest neighbor matching with 0.1 caliper. Assembled using the R package MatchIt, the method computed a distance between each unit, then assigned each unit a control unit. Rather than optimizing an overall criterion, no effort was made to select matches based on the effects of possible future matches. In order to compare clinical outcomes between the two groups, we used appropriate statistical tests after matching. In conducting propensity score matching, we used the variables, including age, gender, BMI, COBB degree, preoperative hemoglobin level, TXA use, smoking and alcohol status, comorbidities such as hypertension and diabetes, preoperative albumin level, ASA classification, respiratory diseases, and the number of fused segments.

In this study, we used multivariate conditional logistic regression to evaluate the possible relationship between intraoperative allogeneic blood transfusion and postoperative PPCs, while controlling for baseline characteristics and known PPC risk factors (17, 23). Influence was assessed in terms of the Risk Ratio (RR) and associated 95% confidence interval (CI). Logistic regression was used to perform subgroup analyses, which were plotted in a forest plot. The possibility of a nonlinear relationship between change in operation time and PPC was explored using a logistic regression model incorporating RCS (Restricted Cubic Split). In our study, all statistical analyses were performed using R software, version 4.2.2.

### Statistical power

Group sample sizes of 161 in group 1 and 161 in group 2 achieve 82.333% power to detect a difference between the group proportions of 0.15. The proportion in group 1 (Transfusion group) is assumed to be 0.26 under the null hypothesis and 0.41 under the alternative hypothesis. The proportion in group 2 (Non-transfusion group) is 0.26. The test statistic used is the two-sided Z-Test with unpooled variance. The significance level of the test is 0.05. We used PASS 21 (Kaysville, Utah, USA.).

## Results

**Figure 1:**
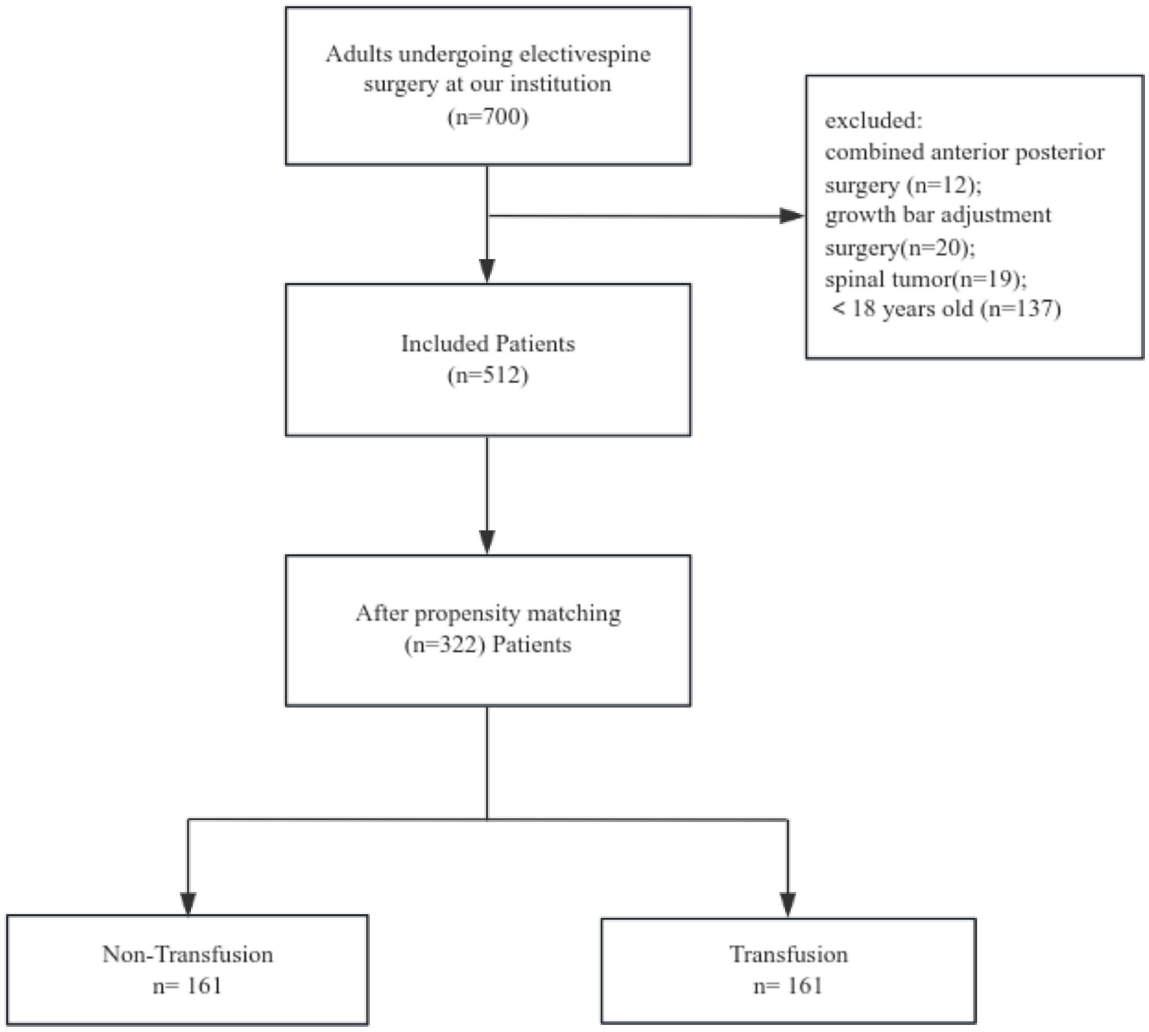
Patient enrolment flowchart.

In this study, an initial cohort of 700 patients was subjected to screening, resulting in the selection of 512 individuals. Propensity score matching was conducted to ensure comparability in terms of relevant characteristics, leading to the inclusion of 161 pairs of patients. Each pair consisted of one patient who received at least 1 unit of red blood cell transfusion and another patient who did not receive any transfusion (Figure 1). Among these paired patients, the occurrence of PPCs was found to be 41% in the transfusion group, whereas it was 27% in the non-transfusion group. In total, approximately 30% of patients experienced at least one postoperative pulmonary complication both prior to and subsequent to the pairing, encompassing pleural effusion, pulmonary atelectasis, pneumonia symptoms, and haemopneumothorax (Table 1).

**Table 1:** Baseline covariates before and after matching.

Character	Level	Before matching			After matching		
		No^a^	Yes^a^	SMD^△^	No^a^	Yes^a^	SMD^△^
*n*		294	218		161	161	
Age (years)		60.02 (15.93)	53.75 (19.37)	−0.323	58.83 (17.73)	57.60 (17.41)	−0.064
Gender	Male	79 (26.9)	39 (17.9)	−0.234	29 (18.0)	33 (20.5)	0.065
	Female	215 (73.1)	179 (82.1)	0.234	132 (82.0)	128 (79.5)	−0.065
BMI (kg/m^2^)		23.15 (3.74)	21.99 (3.72)	−0.314	22.42 (3.83)	22.68 (3.66)	0.070
Smoking	No	270 (91.8)	207 (95.0)	0.142	154 (95.7)	153 (95.0)	−0.028
	Yes	24 (8.2)	11 (5.0)	−0.142	7 (4.3)	8 (5.0)	0.028
Alcohol	No	265 (90.1)	207 (95.0)	0.220	152 (94.4)	151 (93.8)	−0.028
	Yes	29 (9.9)	11 (5.0)	−0.220	9 (5.6)	10 (6.2)	0.028
Hypertension	No	197 (67.0)	164 (75.2)	0.190	111 (68.9)	116 (72.0)	0.072
	Yes	97 (33.0)	54 (24.8)	−0.190	50 (31.1)	45 (28.0)	−0.072
Diabetes	No	266 (90.5)	205 (94.0)	0.150	150 (93.2)	151 (93.8)	0.026
	Yes	28 (9.5)	13 (6.0)	−0.150	11 (6.8)	10 (6.2)	−0.026
Respiratory diseases	No	277 (94.2)	203 (93.1)	−0.043	150 (93.2)	148 (91.9)	−0.049
	Yes	17 (5.8)	15 (6.9)	0.043	11 (6.8)	13 (8.1)	0.049
Preoperative HB (g/L)		128.0 (16.37)	122.6 (15.77)	−0.346	123.5 (16.27)	124.7 (14.89)	0.078
Preoperative ALB (g/L)		40.12 (4.40)	39.77 (4.06)	−0.088	39.69 (4.66)	39.82 (4.14)	0.032
ASA	1	10 (3.4)	8 (3.7)	0.014	6 (3.7)	6 (3.7)	0.000
	2	257 (87.4)	191 (87.6)	0.006	139 (86.3)	144 (89.4)	0.094
	3	26 (8.8)	19 (8.7)	−0.005	16 (9.9)	11 (6.8)	−0.110
	4	1 (0.3)	0 (0.0)	−0.077	0 (0.0)	0 (0.0)	0.000
COBB (degree)		32.1 (10.57)	40.8 (15.06)	0.577	35.8 (9.65)	36.5 (11.81)	0.046
Fusion segments (n)		4.6 (2.3)	5.5 (2.2)	0.334	5.7 (2.7)	5.5 (2.3)	0.021
TXA (%)	No	203 (69.0)	122 (56.0)	−0.264	99 (61.5)	99 (61.5)	0.000
	Yes	91 (31.0)	96 (44.0)	0.264	62 (38.5)	62 (38.5)	0.000
Pneumonia	No	290 (98.6%)	211 (96.8%)		155 (96.3%)	157 (97.5%)	
	Yes	4 (1.4%)	7 (3.2%)		6 (3.7%)	4 (2.5%)	
Atelectasis	No	280 (95.2%)	197 (90.4%)		149 (92.5%)	152 (94.4%)	
	Yes	14 (4.8%)	21 (9.6%)		12 (7.5%)	9 (5.6%)	
Pleural effusion	No	232 (78.9%)	135 (61.9%)		97 (60.2%)	121 (75.2%)	
	Yes	62 (21.1%)	83 (38.1%)		64 (39.8%)	40 (24.8%)	
Pneumothorax	No	293 (99.7%)	214 (98.2%)		159 (98.8%)	160 (99.4%)	
	Yes	1 (0.3%)	4 (1.8%)		2 (1.2%)	1 (0.6%)	
PPCs	No	228 (77.6%)	131 (60.1%)		95 (59%)	118 (73.3%)	
	Yes	66 (22.4%)	87 (39.9%)		66 (41%)	43 (26.7%)	

^a^Continuous variables are expressed as the mean ± standard deviation, the median (interquartile range), or the number of patients *n* (%). ^△^Standardized Mean Difference, TXA, tranexamic acid; BMI, body mass index; ASA, American society of Aneshesiologists (ASA) physical status classification system; COBB, a measure of the angle of lateral curvature, is used to assess the severity of scoliosis; HB, hemoglobin; ALB, albumin; PPCs, postoperative pulmonary complications. NRS, numerical rating scale.

Following propensity score matching, the SMD values were all below 0.1, indicating that the differences between groups were minimal. This suggests that the baseline characteristics were well-balanced, supporting the validity of our statistical analysis. Our objective was to assess the disparity in the occurrence of PPCs between the two cohorts. The primary outcome of interest was PPCs, and we employed chi-square tests and logistic regression analyses to compare the findings across the two groups. The chi-square test yielded risk ratio (RR 1.53, 95% CI 1.12–2.11)values, with a *p*-value of 0.007. Subsequently, we compared the relationship between the volume of blood transfused and PPCs and found a risk ratio (RR 1.40, 95% CI 1.04–1.89) when the volume of blood transfused was greater than 400 mL, with a chi-square test (*p* = 0.030) (Table 2). To assess the robustness of our findings, we conducted treatment effectiveness comparisons for both pre-matching and post-matching data. Additionally, we performed sensitivity analyses to further examine the reliability of our findings (Supplementary Tables 1, 2).

**Table 2:** Differential comparison and logistic regression between different blood transfusion groups in pulmonary complications.

Character	Non-transfusion, *n* = 161^1^	Transfusion, *n* = 161^1^	Odds Ratio, OR (95% CI)	*p* ^2^	Transfusion < 400 ml, *n* = 195^1^	Transfusion ≥ 400 ml, *n* = 127^1^	Odds Ratio, OR (95% CI)	*p* ^2^
Respiratory complication			1.91 (1.19–3.05)	0.007			1.68 (1.05–2.68)	0.030
No	118 (73%)	95 (59%)			138 (71%)	75 (59%)		
Yes	43 (27%)	66 (41%)			57 (29%)	52 (41%)		

^1^n (%).

^2^Pearson’s Chi-squared test. CI = Confidence Interval.

Transfusion includes only allogeneic blood.

The covariates included age, sex, ASA classification, Cobb angle, and the number of fused segments.

**Figure 2:**
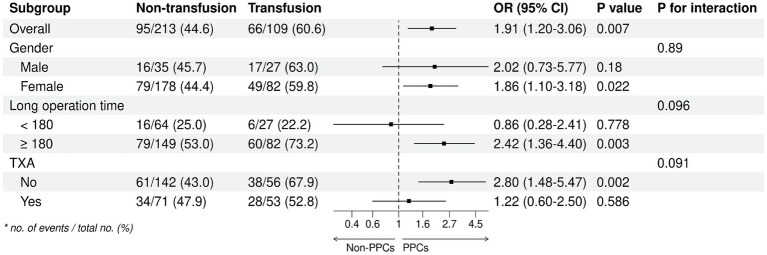
Subgroup analysis. TXA: tranexamic acid.

**Figure 3:**
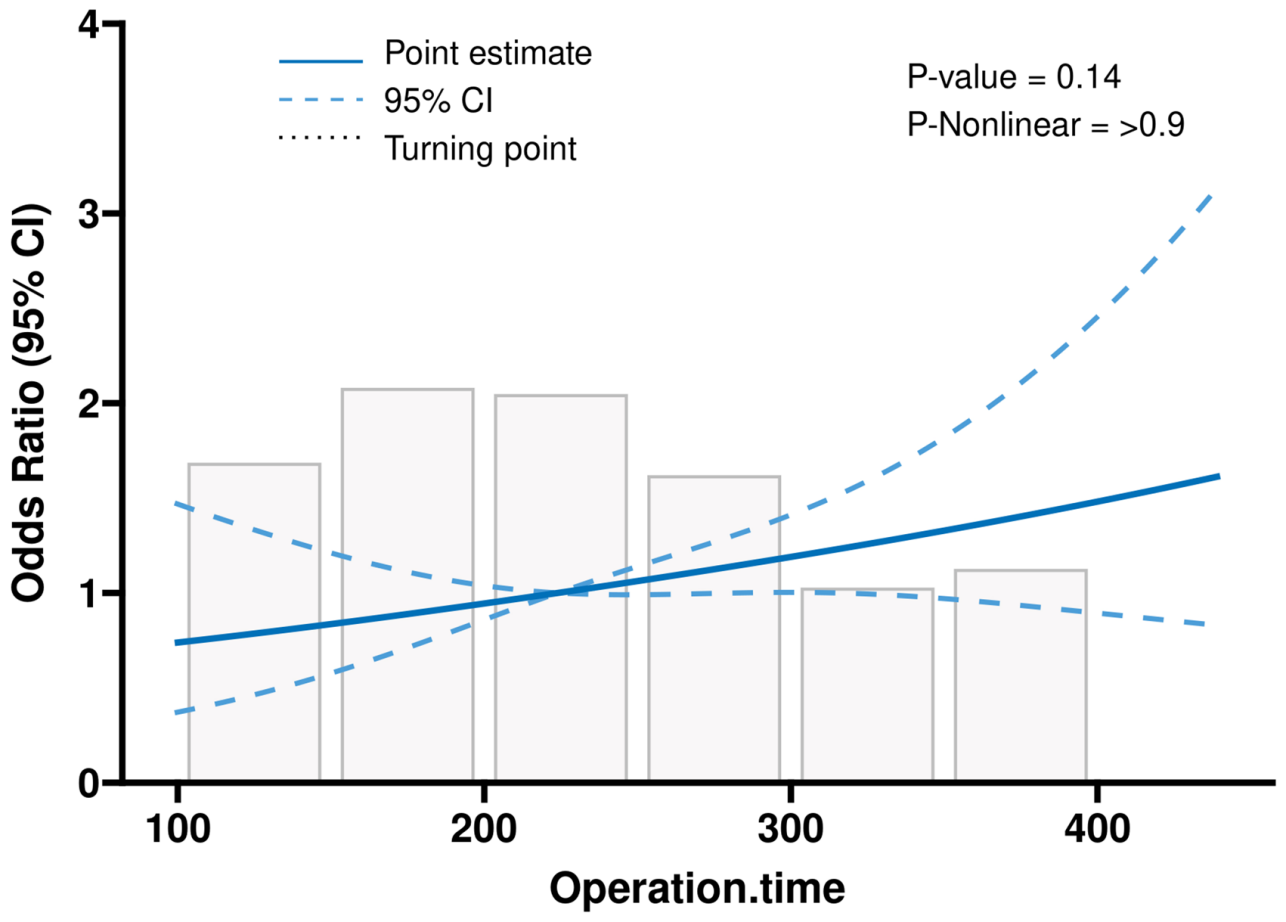
Linear relationships between operation time and the postoperative outcome, depicted using an RCS-based one-way logistic regression model.

We analysed the subgroups according to different parameters of the primary endpoint (Figure 2). The results of our analyses indicate a higher likelihood of postoperative pulmonary complications (PPC) in female subjects, subjects with a surgical duration exceeding 3 h, and subjects who did not administer tranexamic acid (TXA) during the operation. Subsequently, an investigation was conducted to examine the correlation between the duration of surgical procedures and the occurrence of PPCs, yielding a *p*-value of 0.14. This outcome suggests a positive linear relationship, wherein an increase in surgical duration corresponds to an elevated likelihood of PPCs (Figure 3). In addition, we found that blood transfusion was moderately associated with the duration of surgery (Transfusion Volume *r* = 0.537, 95% CI 0.455–0.611, *p* < 0.001; Transfusion *r* = 0.456, 95% CI 0.365–0.539, *p* < 0.001) (Supplementary Table 3; Supplementary Figures 1, 2).

## Discussion

We found in this retrospective study that intraoperative allogeneic red blood cell transfusion significantly increased risk of PPCs. This effect of transfusion was dose-dependent, with the risk of PPCs rising steeply when transfusion volume exceeded 400 mL. The results of our analyses indicate that the occurrence of PPC was significantly higher among female subjects, subjects with an operative time exceeding 3 h, and subjects who did not utilize TXA during the intraoperative period. As the duration of surgical procedures lengthens, the likelihood of experiencing PPCs escalates.

To this day, there is a lack of definitive evidence from randomized controlled trials (RCTs) regarding the correlation between allogeneic blood transfusion and the incidence of PPCs in orthopaedic spinal surgery. The utilization of propensity score matching aids in equalizing the influence of known confounding variables among different groups (24). By employing propensity score matching to pair individuals with similar scores, the potential bias arising from these variables is mitigated. The data presented in Table 1 demonstrates a noteworthy decrease in standardized mean differences (SMD) subsequent to matching, indicating that propensity score matching effectively enhances the equilibrium of covariates between the two treatment groups. This enhanced equilibrium enhances the reliability of treatment effect comparisons by minimizing the potential influence of confounding factors. Furthermore, the inclusion of sensitivity analyses can enhance the dependability of research findings by evaluating the resilience of various matching strategies or methodologies to the outcomes of the study, akin to the sensitivity analyses employed in randomized controlled trials (RCTs) (25).

Gaining comprehension of the correlation between blood transfusions during surgical procedures and subsequent PPCs proves highly advantageous in the realm of academia (26). Such understanding facilitates the development of strategies aimed at reducing blood utilization and identifying alternative preventive measures for postoperative complications (27). Consequently, patients’ recovery outcomes are enhanced, and their associated risks are diminished (28, 29). Should a causal relationship between blood transfusions and these complications be established, it has the potential to revolutionize the administration of blood transfusions in surgical settings (30, 31). This paradigm shift may engender improved surgical techniques and patient management approaches that mitigate the necessity for extensive blood transfusions. This information could be utilized by medical practitioners to enhance surgical planning (32). Acquiring knowledge of these factors enables us to assess the potential risks associated with scoliosis patients undergoing surgery. Furthermore, it may serve as a catalyst for further investigations aimed at developing interventions that mitigate these complications, ultimately enhancing the safety of surgical procedures in the long run.

Following scoliosis correction surgery, the prevailing pulmonary complications typically encompass atelectasis, pneumonia, pleural effusion, and respiratory insufficiency, whether systemic or specific in nature (33). These complications commonly arise due to alterations in lung function subsequent to surgery, modifications in breathing patterns, diminished mobility, and the impact of anesthesia. To mitigate these risks and facilitate a more seamless recuperation process for patients undergoing scoliosis correction surgery, preventive measures such as early mobilization, respiratory exercises, and vigilant post-operative care have been devised (34, 35). This study primarily examines prevalent complications, namely atelectasis, pneumonia, and pleural effusion, which can be ameliorated through proactive postoperative intervention (36). It is important to note that respiratory failure, although not encompassed within the study’s postoperative observation period, may manifest subsequently during the recovery phase or following discharge, thus eluding inclusion within the study’s temporal scope (37). On the other hand, proactive treatment measures have frequently been implemented prior to the onset of respiratory failure, leading to a reduced incidence of such occurrences. Subsequently, heightened focus will be directed toward the emergence of severe complications, thereby offering novel insights for future investigations (38).

Our study found that the incidence of postoperative pulmonary complications (PPCs) in patients undergoing scoliosis correction surgery ranged from 27 to 40%, which is consistent with existing literature. Wang Y et al. (17) reported an overall PPCs incidence of 40.8%, with pleural effusion occurring in 47.6% and pneumonia in 40.2%, which is similar to our findings. Wu L et al. (4) reported a pleural effusion rate of 75.6% and a pneumonia rate of 53.3%. Kang GR et al. (39) reported a PPCs incidence ranging from 18 to 66.7%. Additionally, Soroceanu A et al. (40) highlighted that 27% of patients undergoing spinal surgery experience at least one postoperative complication. Overall, our findings align with the literature, further confirming the high risk of PPCs in patients undergoing scoliosis correction surgery.

The present study reveals a positive correlation between the duration of surgical procedures and the incidence of PPCs among patients (41). Prolonged surgeries are primarily influenced by factors such as pre-existing severe scoliosis, the extent of spinal segments requiring treatment, and the occurrence of surgical trauma (42). Furthermore, lengthier surgical interventions are frequently associated with an elevated probability of necessitating a blood transfusion and receiving a greater volume of blood during the procedure (43). This augmented transfusion requirement is intricately connected to deteriorating patient conditions and potentially exacerbates the complexity of the surgical intervention. In order to mitigate these risks, it is imperative to effectively manage anemia, safeguard blood integrity, and adhere to surgical protocols aimed at minimizing surgical duration and diminishing the likelihood of complications.

## Limitation

Due to the retrospective nature of our study, caution is warranted in interpreting our findings. Despite the limited sample size, the data at our disposal enabled us to detect significant disparities in crucial outcomes. Although unmeasured variables may potentially influence our findings, the utilization of propensity score matching assists in mitigating the impact of these unknown factors. This technique effectively equalizes the measured variables, thereby reducing the likelihood of substantial alterations in our results due to unmeasured variables. Our study offers preliminary insights suggesting a potential association between the administration of allogeneic blood transfusion during spinal surgeries and an increased likelihood of postoperative pulmonary complications (PPCs). Should corroborative investigations align with our findings, it implies that reducing the volume of blood transfusions during these procedures may offer a more efficacious approach to enhancing patient outcomes.

Additionally, consistent documentation on the postoperative use of incentive spirometry and mobilization was not available, preventing us from assessing the impact of these preventive measures on pulmonary complications. Future studies could benefit from more detailed tracking of these measures to better explore their effects on outcomes. We also recognize that the risk of pulmonary complications may be influenced by factors such as smoking history and the severity of respiratory conditions. Although we considered variables like Chronic Obstructive Pulmonary Disease (COPD) in our baseline data collection, the low incidence of these conditions in our population limited their impact on the matching process.

## Conclusion

Our findings present a valuable opportunity to enhance surgical techniques, improve patient care, and potentially facilitate the development of novel strategies to mitigate PPCs in scoliosis correction surgery.

## Data Availability

The raw data supporting the conclusions of this article will be made available by the authors, without undue reservation.

## Footnotes

^1^ http://www.equator-network.org/
