# Supplementary material for: Propensity score matching analysis of the relationship between allogeneic blood transfusion and postoperative pulmonary complications in scoliosis correction surgery: a retrospective study
Source: Front Med (Lausanne). 2025 Oct 14;12:1588218. doi: 10.3389/fmed.2025.1588218 (PMC12558844; doi:10.3389/fmed.2025.1588218)
Supplement: Supplementary file 1 [file Data_Sheet_1.docx]

Supplementary Table 1 :Effectiveness Analysis for unmatched data

| **Characteristic** | **no**, N = 294^1^ | **yes**, N = 218^1^ | **p-value**^2^ |
| --- | --- | --- | --- |
| Respiratory complication |  |  | <0.001 |
| no | 228 (78%) | 131 (60%) |  |
| yes | 66 (22%) | 87 (40%) |  |
| Unstratified Response Analysis |  |  |  |
| Risk Difference, RD (%) (95% CI) |  |  | 17.46 (9.40 - 25.52) |
| Risk Ratio, RR (95% CI) |  |  | 1.78 (1.36 - 2.32) |
| Odds Ratio, OR (95% CI) |  |  | 2.29 (1.56 - 3.37) |
| p-value (Chi-squared test) |  |  | <0.0001 |
| p-value (Fisher's exact test) |  |  | <0.0001 |
| ^1^n (%)  ^2^Pearson's Chi-squared test. CI = Confidence Interval  Transfusion includes only allogeneic blood  The covariates included age, sex, ASA classification, Cobb angle, and the number of fused segments.  Abbreviations: OR, odds ratio; CI, confidence interval. | | | |

Supplementary Table 2 Relationship between duration of surgery and postoperative pulmonary complications

| **Characteristic** | **OR per SD**^1^ | **95% CI**^1^ | **p-value** |
| --- | --- | --- | --- |
| Operation time (< 180) | 1.38 | 0.94, 2.08 | 0.10 |
| Operation time (≥ 180) | 1.40 | 1.12, 1.76 | 0.003 |
| ^1^OR = Odds Ratio, CI = Confidence Interval  Supplementary Table 3: Correlation between operative time and blood transfusion and volume, Pearson's correlation coefficient   \|  \|  \| **r** \| **CI** \| **CI low** \| **CI high** \| **t** \| **Df error** \| **p** \| **Method** \| **N Obs** \| \| --- \| --- \| --- \| --- \| --- \| --- \| --- \| --- \| --- \| --- \| --- \| \| Operation Time \| Amount of Transfusion \| 0.537 \| 0.95 \| 0.455 \| 0.611 \| 11.407 \| 320 \| <0.001 \| Pearson correlation \| 322 \| \| Operation Time \| Transfusion \| 0.456 \| 0.95 \| 0.365 \| 0.539 \| 9.185 \| 320 \| <0.001 \| Pearson correlation \| 322 \|   r: Pearson correlation coefficient; CI: Confidence Interval; CI low: Lower bound of CI; CI high: Upper bound of CI; t: t-statistic; Df error: Degrees of Freedom for error; p: p-value; Method: Statistical method used; N Obs: Number of observations. | | | |


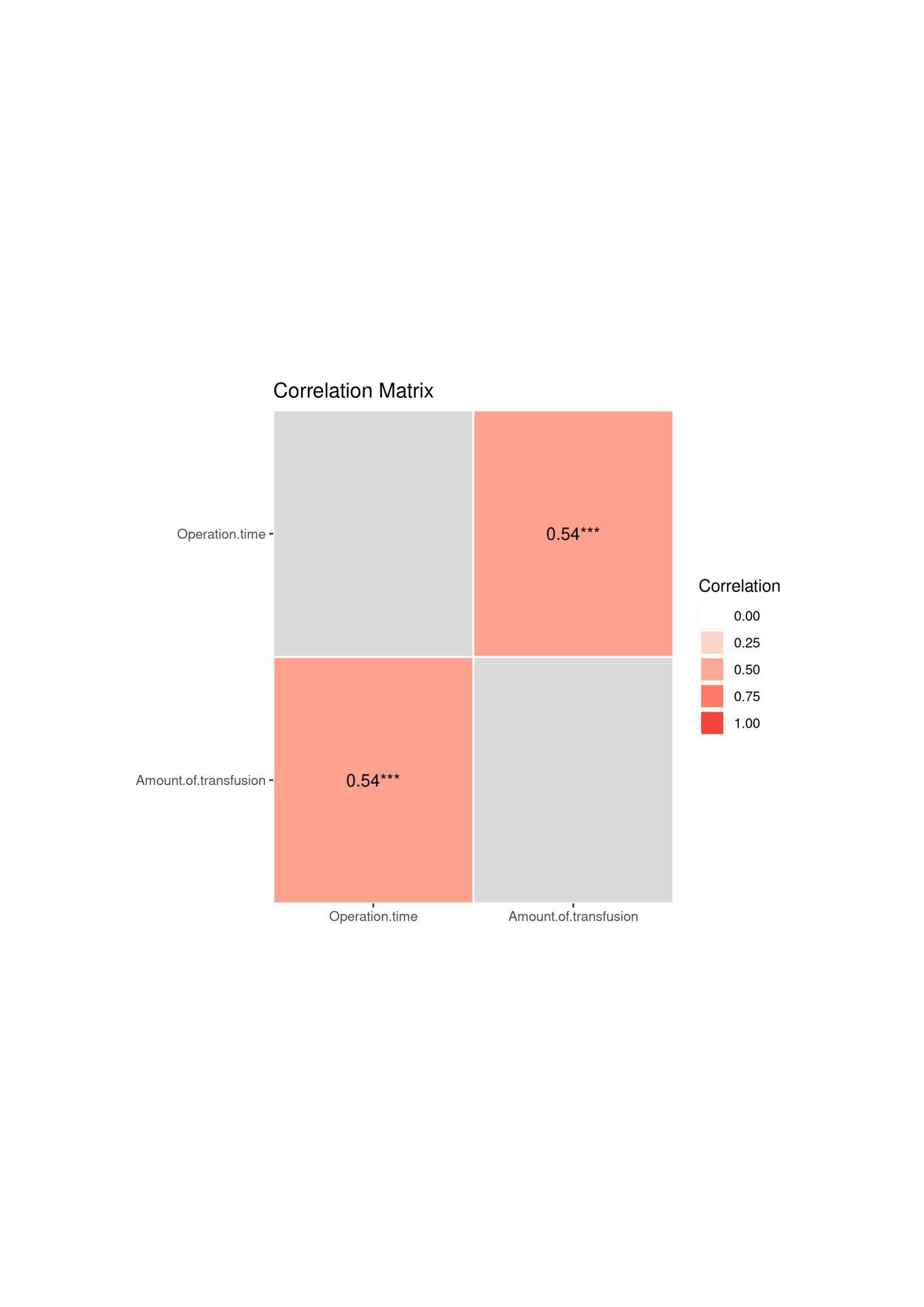


Supplement figure 1. Correlation between operative time and intraoperative blood transfusion volume


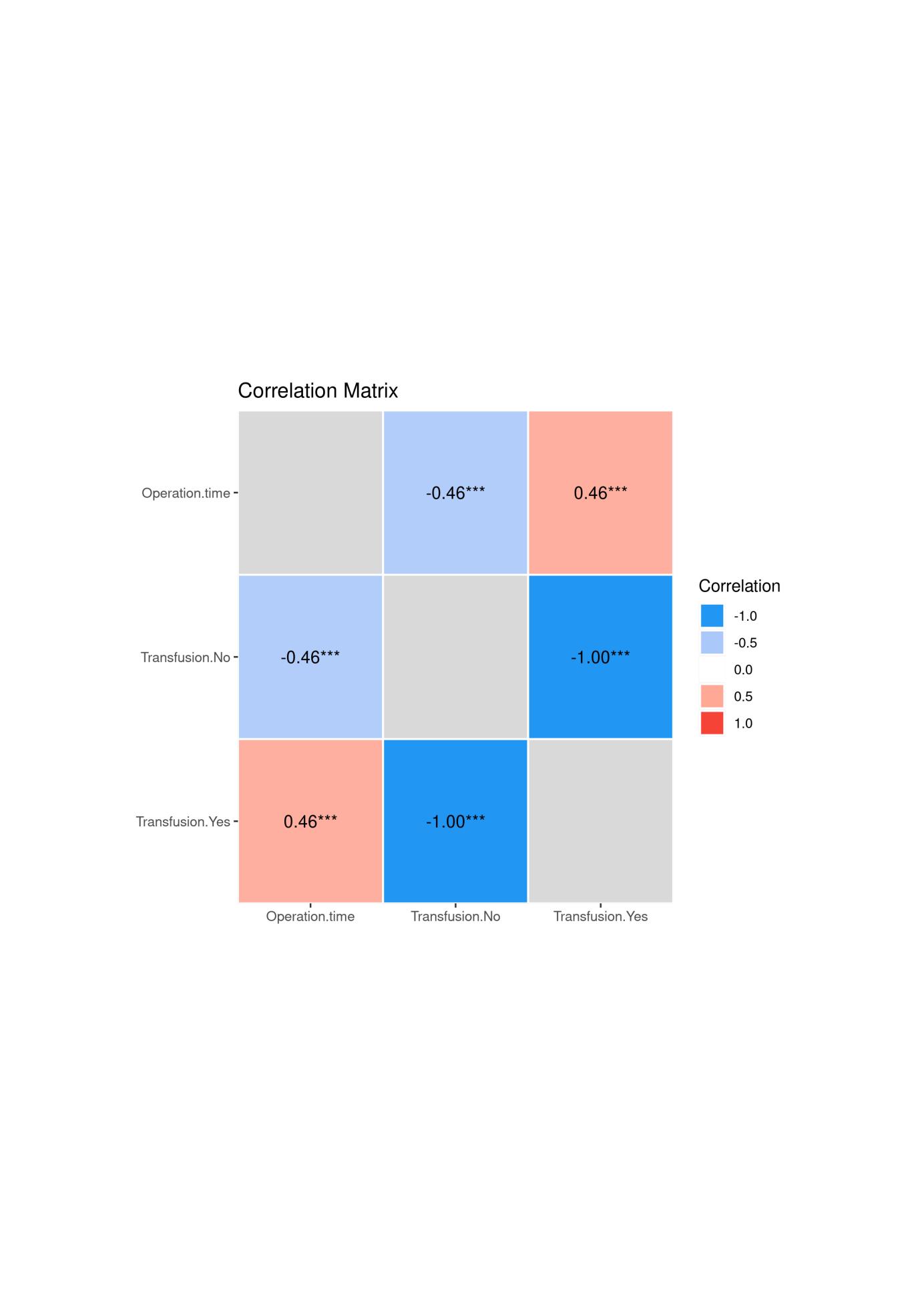


Supplement figure 2. Correlation between operative time and intraoperative blood transfusion
